# Effect of metal implants and metal artifacts on back‐projected two‐dimensional entrance fluence determined by EPID dosimetry

**DOI:** 10.1002/acm2.14115

**Published:** 2023-08-13

**Authors:** Zheng Cao, Xiang Gao, Gongfa Liu, Yuanji Pei

**Affiliations:** ^1^ National Synchrotron Radiation Laboratory University of Science and Technology of China Hefei China; ^2^ Hematology & Oncology Department Hefei First People's Hospital Hefei China

**Keywords:** 2D entrance fluence, back‐projection algorithms, DGRT, EPID, metal artifacts, portal dose image

## Abstract

**Purpose:**

To evaluate the errors caused by metal implants and metal artifacts in the two‐dimensional entrance fluences reconstructed using the back‐projection algorithm based on electronic portal imaging device (EPID) images.

**Methods:**

The EPID in the Varian VitalBeam accelerator was used to acquire portal dose images (PDIs), and then commercial EPID dosimetry software was employed to reconstruct the two‐dimensional entrance fluences based on computed tomography (CT) images of the head phantoms containing interchangeable metal‐free/titanium/aluminum round bars. The metal‐induced errors in the two‐dimensional entrance fluences were evaluated by comparing the γ results and the pixel value errors in the metal‐affected regions. We obtained metal‐artifact‐free CT images by replacing the voxel values of non‐metal inserts with those of metal inserts in metal‐free CT images to evaluate the metal‐artifact‐induced errors.

**Results:**

The γ passing rates (versus PDIs obtained without a phantom in the beam field (PDI_air_), 2%/2 mm) for the back‐projected two‐dimensional entrance fluences of phantoms containing titanium or aluminum (BP_Ti_/BP_Al_) were reduced from 92.4% to 90.5% and 90.6%, respectively, relative to the metal‐free phantom (BP_metal‐free_). Titanium causes more severe metal artifacts in CT images than aluminum, and its removal resulted in a 0.0022 CU (median) reduction in the pixel value of BP_Ti artifact‐free_ relative to BP_Ti_ in the metal‐affected region. Moreover, the mean absolute error (MAE) and root mean square error (RMSE) decreased from 0.0050 CU and 0.0063 CU to 0.0034 CU and 0.0040 CU, respectively (vs. BP_metal‐free_).

**Conclusion:**

Metal implants increase the errors in back‐projected two‐dimensional entrance fluences, and metals with higher electron densities cause more errors. For high‐electron‐density metal implants that produce severe metal artifacts (e.g., titanium), removing metal artifacts from the CT images can improve the accuracy of the two‐dimensional entrance fluences reconstructed by back‐projection algorithms.

## 1 INTRODUCTION

During radiotherapy, anatomy changes, setup errors and accelerator dose output errors can cause the actual absorbed dose in vivo to differ from that calculated by the treatment planning system (TPS) based on the planning computed tomography (CT) images, which can affect patients’ treatment efficacy and safety. To ensure that the tumor target and organs at risk (OARs) receive the planned dose, dose‐guided radiotherapy (DGRT) techniques have been developed to monitor the actual dose received by the patient throughout treatment and to continuously adjust the radiotherapy plan based on the results of the comparisons with the planned dose.^1^, ^2^, ^3^, ^4^, ^5^, ^6^

In vivo three‐dimensional (3D) dose reconstruction during treatment is the most critical DGRT technique. One promising approach involves the combination of back‐projection and forward‐calculation algorithms based on electronic portal imaging device (EPID) images.^7^ During the patient's radiotherapy, EPID images are acquired first. Then, the patient's CT images or cone beam computed tomography (CBCT)‐based synthetic CT images are input into a back‐projection algorithm to reconstructed the two‐dimensional (2D) entrance fluence. Finally, the in vivo 3D dose distribution for the patient is obtained using forward‐calculation algorithms via the TPS based on the reconstructed 2D entrance fluence. This novel radiotherapy technique allows the estimation of the patient's in vivo dose distribution for each treatment fraction without additional radiation exposure. Despite the challenges of obtaining accurate results, this technique has the potential to improve radiotherapy efficacy and reduce adverse effects by allowing timely adjustment of the treatment plan, and extensive research has been conducted on this topic. Hansen et al. obtained the primary fluence distribution within patients by back‐projecting the EPID images to CT images and then convolved the primary fluence distribution with the dose deposition kernel to calculate the in vivo 3D dose distribution, achieving errors of no more than 2% compared to measurements obtained by film and thermoluminescence dosimeters.^8^ Jarry et al. utilized the Monte Carlo algorithm to back‐project the EPID images and reconstruct the in vivo dose distribution.^9^ The reconstructed dose distribution of the intensity modulated radiotherapy (IMRT) plans achieved a γ passing rate (5%/3 mm) of 90%−96% relative to the Monte Carlo calculation, and a γ passing rate of 80%−85% relative to the film measurements. Van Uytven et al. developed a model‐based 3D in vivo dose reconstruction algorithm that combined the back projection algorithm of EPID images with a forward‐calculation algorithm.^7^ The algorithm achieved high chi pass rates (2%/2 mm) of 97.6%–99.7% for volumetric modulated arc therapy (VMAT) and dynamic IMRT plans.

The CT values in the patient's CT images can be converted to relative electron density (RED) distributions using a CT value‐RED calibration curve, which is used as the fundamental data for back‐projection calculations based on the EPID images. This enables the derivation of the 2D entrance fluence and forms the basis for utilizing forward‐calculation algorithms to acquire 3D in vivo dose distributions. During a CT scan, X‐rays passing through metal implants such as dentures and hip replacements can produce inaccurate X‐ray projections due to beam hardening, scattering, photon starvation, noise enhancement, and volume effects.^10^, ^11^, ^12^, ^13^, ^14^ As a result, the reconstructed image may show bright and dark stripes, which can affect the accuracy of the RED distribution information obtained from the scan. In our previous study, we showed that metal artifacts caused by metal dentures can greatly reduce the dose accuracy in the OARs and nearby planning target volumes (PTVs).^15^ Metals and metal artifacts in CT images can also affect the accuracy of the 2D entrance fluence reconstructed by back‐projection algorithms and EPID images. However, to our knowledge, no studies have been conducted on this topic.

In this study, we acquired both metal‐containing and metal‐free CT images, as well as the corresponding EPID images of an anthropomorphic head phantom, by replacing its cylindrical inserts. To assess the impact of metal artifacts, we replaced the voxel values of the non‐metal inserts in the metal‐free CT images with those of the metal inserts. This allowed us to obtain CT images that were completely free of metal artifacts. We utilized a commercial in vivo EPID dosimetry software to perform back‐projection calculations based on various EPID images using different CT images. By comparing the γ results of the 2D entrance fluence for each group, as well as the pixel value differences in the metal‐affected regions, we determined the impact of different metal types and their metal artifacts on the accuracy of the back‐projection calculation, and elucidated the underlying principles.

## 2 MATERIALS AND METHODS

### 2.1 Research process

**FIGURE 1:**
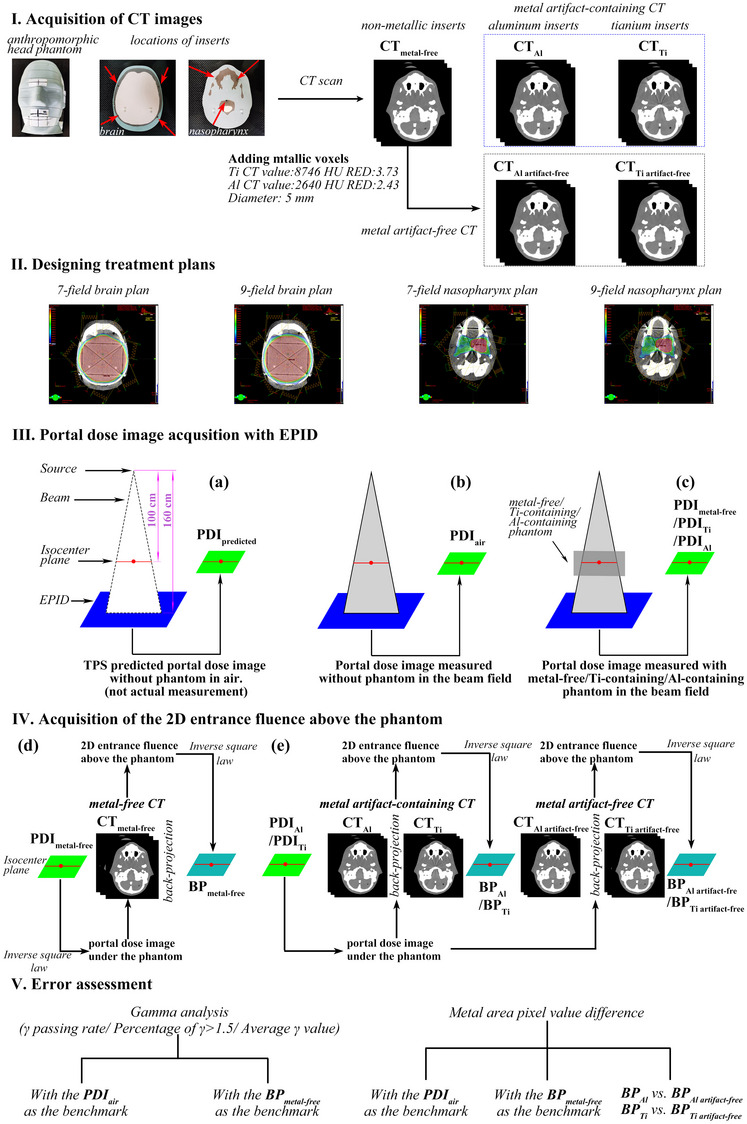
Schematic diagram. The process of this research can be divided into five parts: I. Acquisition of CT images; II. Designing of treatment plans; III. Acquisition of portal dose images with the EPID system; IV. Acquisition of the 2D entrance fluences above the phantom; and V. Error assessment.

As shown in Figure 1, we conducted this study in five steps. First, we acquired the metal‐free phantom, Ti‐ and Al‐containing phantoms and their corresponding CT images (CT_metal‐free_, CT_Ti_, CT_Al_) by replacing the inserts in the anthropomorphic head phantom. The images of the latter two phantoms contained metal artifacts (Figure 1.I). We also obtained the CT images of the Ti‐containing and Al‐containing phantoms without metal artifacts by replacing the voxel values of the non‐metal inserts in CT_metal‐free_ with the voxel values of the corresponding metals (Figure 1.I, Figure S1). Second, we designed separate treatment plans for the brain and nasopharynx (Figure 1.II). Third, we obtained the portal dose image (PDI) predicted by the TPS without a phantom in the beam field (PDI_predicted_), the measured PDI without a phantom in the beam field (PDI_air_), and the measured PDIs with metal‐free, Ti‐containing and Al‐containing phantoms in the beam field (PDI_metal‐free_/PDI_Ti_/PDI_Al_) based on the EPID images (Figure 1.III). Fourth, we calculated the 2D entrance fluences in the isocenter plane based on the measured PDI and the corresponding CT images using the back‐projection algorithm and the inverse square law (Figure 1.IV). Fifth, we analyzed the effect of different metal materials on the back‐projection accuracy and the effect of metal artifact reduction (MAR) on the error using γ analysis and pixel value error comparison in the metal‐affected region (Figure 1.V).

### 2.2 Anthropomorphic phantom

We employed a heterogeneous anthropomorphic head phantom, the CIRS ATOM 701‐B dosimetry phantom (Figure 1.I), as a substitute for a live patient's head in our study. This particular phantom is outfitted with 5 mm diameter cylindrical inserts that can be interchanged at various locations. To simulate human heads with different metal implants, we replaced the inserts with titanium and aluminum alloy counterparts (Figure 1.I).

### 2.3 Acquisition and preprocessing of CT images

The CT images were captured with a GE Discovery RT helical CT scanner (General Electric Company, MA, USA), employing a tube voltage of 120 kV, a slice thickness of 2.5 mm, an image resolution of 0.572 mm × 0.572 mm, and a depth of 16 bits. To eliminate errors caused by air noise in the images during the back‐projection calculation, voxel values beyond the skin were designated as −1000 HU.

The REDs for the titanium and aluminum alloys were 3.73 and 2.43, respectively. The metal CT values of these alloys were derived based on CT images containing the corresponding titanium and aluminum alloys, with CT values of 8746 HU and 2640 HU, respectively. High RED value metals, such as titanium, induce severe metal artifacts in CT images that degrade image quality and accuracy. Existing commercial MAR algorithms (e.g., GE's SmartMAR, Philips’ O‐MAR, and Siemens’ iMAR) cannot completely eliminate these artifacts and may introduce new artifacts (Figure S2). To obtain the true RED distribution of the metal‐containing phantom, we simulated artifact‐free CT images by replacing the voxel values of the non‐metal inserts in the CT images of the metal‐free phantom with those of titanium and aluminum, respectively (Figure S1). These images (CT_Ti artifact‐free_ and CT_Al artifact‐free_) contained titanium and aluminum voxels, respectively, without any metal artifacts.

### 2.4 Designing radiotherapy treatment plans

Based on the CT_metal‐free_ images, two sets of dynamic IMRT plans with seven and nine beam fields were designed for the brain and nasopharynx using the TPS (Eclipse 15.6, AXB15.6.06 algorithm) (Figure 1.II). The beam angles were averaged over 360°. The brain clinical target volume (CTV) was outlined as a 2.5 cm in diameter circle. The PTVs were obtained by externalizing the CTV contours by 4 cm, covering approximately the entire brain tissue in each slice to ensure that each beam field passed through all metal rods, thus creating a uniform dose distribution. The CTV and gross target volume (GTV) of the nasopharyngeal carcinoma were outlined to mimic a real patient's target volume. The PTV and planning gross target volume (PTV‐G) were obtained by externalizing the CTV and GTV contours by 5 mm each. The OAR contours were outlined by referencing a real patient's anatomy. For the seven‐field and nine‐field brain plans, the prescribed dose for PTV was 6000 cGy in 30 treatment fractions. For the seven‐field and nine‐field nasopharynx plans, the prescribed doses for the PTV and PTV‐G were 6006 cGy and 7029 cGy in 33 treatment fractions, respectively. These plans were replicated with the same multileaf collimator (MLC) and monitor unit (MU) parameters for CT_Ti_, CT_Ti artifact‐free_, CT_Al_, and CT_Al artifact‐free_.

### 2.5 EPID equipment and imaging dosimetry calibration procedures

For this study, we used the Varian VitalBeam linear accelerator (Varian Medical Systems, CA, USA) equipped with 60 pairs of MLCs and an a‐Si 1200 portal imager to acquire EPID images. The a‐Si 1200 portal imager measures 40 ×  40 cm^2^, and the EPID images have a size of 1190 ×  1190 pixels with a resolution of 0.336 mm. The EPID detector was fixed at a distance of 160 cm from the source for this study.

To ensure the accuracy of the EPID images, it is necessary to calibrate the dose of the imaging system at the accelerator workstation following the Varian Technical Reference Guide. The calibration process includes five sequential steps, dark field, flood field, pixel correction, beam profile, and dose normalization, which are all carried out in the MV image calibration mode of the workstation. Throughout the calibration process, the flat panel detector must be maintained at a fixed distance of 160 cm from the source, and the X‐ray energy should be set to 6 MV. For the beam profile calibration step, a reference is established using a 40 ×  40 cm^2^ field at a depth of 5 cm in water. In portal dosimetry, it is common to use the convention of 1 MU delivered at 100 cm to a 10 ×  10 cm^2^ field. Since the detector was located 160 cm from the source in this study, a target MU of 1 was used in the dose normalization step, and the reference dose was set to 0.391 calibrated unit (CU) according to the inverse square law.

### 2.6 Acquisition of the PDIs

In this study, the EPID detector was positioned at a source‐to‐detector distance (SDD) of 160 cm. For consistent comparison, all the PDI images and back‐projected 2D entrance fluences were converted to the isocenter plane (SDD = 100 cm) with a size of 1190× 1190 pixels and a resolution of 0.21 mm using the inverse square law.

Figure 1.III illustrates the process of obtaining different PDIs. First, the TPS calculates the predicted PDI (PDI_predicted_) based on the treatment plan parameters without any phantom in the beam field (Figure 1.IIIa). Then, after delivering the plan, the EPID acquires the actual PDI in air (PDI_air_) without any phantom in the beam field (Figure 1.IIIb), and the PDIs with three different phantoms in the beam field, the metal‐free (PDI_metal‐free_) phantom, Ti‐containing (PDI_Ti_) phantom, and Al‐containing phantom (PDI_Al_) (Figure 1.IIIc).

### 2.7 Acquisition of the 2D entrance fluence above the phantom

We used the commercial in vivo EPID dosimetry software called KylinRay‐Dose4D (SuperAccuracy Science & Technology Co., Ltd, Nanjing, China) to implement the back‐projection algorithm and reconstruct the 2D entrance fluence. The software is based on the following principle and procedure. The photons received on the flat plane of the EPID are split into primary and scattered rays. The gradients at the field edge differ significantly, resulting in a more pronounced differences in the grayscale distribution gradients of the primary and scattered rays at the field edges in the EPID images. Based on this feature, the software calculates the scattered ray distribution at the EPID flat plane and subtracts the scattered ray grayscale image from the total grayscale image to obtain the grayscale image generated by the primary rays. Then, the primary ray intensity distribution at the EPID top surface is obtained by deconvolution. Next, the equivalent water thickness of the photon penetration phantom is obtained based on the CT information, and the photon energy spectrum and the mass attenuation coefficient of the substance are combined to obtain the photon penetration phantom attenuation coefficient using a fast Monte Carlo calculation method based on GPU parallelism.^16^ Finally, 2D entrance fluence at the human or phantom surface is reconstructed by applying the inverse square law and correcting for the photon beam attenuation.^17^, ^18^ The calculation grid size of KylinRay‐Dose4D corresponded to a PDI size of 1190 ×  1190 pixels and a resolution of 0.21 mm at the isocenter plane.

In clinical applications, it is desirable to use online CT images of the patients (or phantoms) acquired before each treatment for back‐projection calculations rather than the planning CT images obtained at the time of setup because the patient's anatomical structures and positions may vary between the initial setup and each treatment. However, our radiotherapy center has only one CBCT scanner that can provide online images. Although we previously developed a method to convert CBCT images into synthetic CT images with reduced metal artifacts, the RED information in the synthetic CT images may not be completely accurate, which may introduce additional errors in the back‐projection calculation.^15^, ^19^ In this study, we used a simulated phantom with a rigid structure and no anatomical variations. The phantom contained only the head region, which was easy to set up, and the setup error in all directions were less than 1 mm. Therefore, we assumed that using the planning CT image of the phantom instead of the online image in the back‐projection calculation was sufficiently accurate.

The 2D entrance fluences were reconstructed from different combinations of PDIs and CT images (Figure 1.IV). We denoted the 2D entrance fluences reconstructed based on PDI_metal‐free_ and CT_metal‐free_ as BP_metal‐free_. We denoted the 2D entrance fluences reconstructed based on PDI_Ti_/PDI_Al_ and CT_Ti_/CT_Al_ (with metal artifacts) as BP_Ti_/BP_Al_. Finally, we denoted the 2D entrance fluences reconstructed based on PDI_Ti_/PDI_Al_ and CT_Ti artifact‐free_/CT_Al artifact‐free_ (without metal artifacts) were referred to as BP_Ti artifact‐free_/BP_Al artifact‐free_.

### 2.8 Error assessment

γ analysis is a widely used approach to assess the accuracy of dose distributions. The methodology of γ analysis is explained in detail in the TG‐218 report.^20^ We conducted γ analysis based on an external 1 cm region of the MLC complete irradiation area outline (MLC CIAO) for each field corresponding to different PDIs and back‐projected 2D entrance fluences using Varian's portal dosimetry (PD) system. The PD system's improved γ evaluation calculation algorithm, absolute normalization mode, and global γ evaluation with a threshold of 10% were employed in the analysis. To determine the γ passing rate, mean γ value, and percentage of γ values greater than 1.5, we utilized three different criteria: 3%/3 mm, 3%/2 mm, and 2%/2 mm.

We also evaluated the pixel‐level differences in the back‐projected 2D entrance fluences in the metal‐affected regions for each group. Our analysis included three comparisons: (1) We used PDI_air_ as a reference to compare the differences among all back‐projected 2D entrance fluences; (2) we used BP_metal‐free_ as a reference to compare the effects of different metal types on the back‐projection accuracy and to evaluate the effect of MAR in CT images on error reduction; and (3) we directly compared the back‐projected 2D entrance fluences (BP_Ti/Al_ and BP_Ti artifact‐free/Al artifact‐free_) obtained before and after MAR in CT images and quantified the differences in the metal‐affected region. The results of these comparisons are presented as violin plots, and we calculated the mean absolute error (MAE) and root mean square error (RMSE) for the pixel value differences in the first two comparisons.

(1) $$MAE\;\left(I_{1},I_{2}\right)=\frac{1}{n}\;\sum_{i\;=\;1}^{n}\left|I_{1,i}-I_{2,i}\right|\;$$

(2) $$RMSE\;\left(I_{1},I_{2}\right)=\sqrt{\frac{1}{n}\sum_{i=1}^{n}{\left(I_{1,i}-\;I_{2,i}\right)}^{2}}\;$$

In Equations (1) and (2), *I*_1_ and *I*_2_ denote the two images to be compared, and $I_{1,i}$ and $I_{2,i}$ denote to the CU values of the i‐th pixel in images *I*_1_ and *I*_2_, respectively.

We calculated the difference between the PDI_air_ and PDI_Ti_/PDI_Al_ to identify metal‐affected regions in the back‐projected 2D entrance fluences. X‐rays passing through metal inserts are subject to higher attenuation, resulting in lower pixel values in metal‐affected regions than in non‐metal regions in the difference image. To determine the metal‐affected regions, we calculated the percentile of pixel values in the difference images. After comparing the results, we determined that pixels with values below the third percentile should be identified as metal‐affected regions because most of these pixels were within the area affected by metal in the back‐projected 2D entrance fluences.

We used IBM SPSS Statistics software^21^ to analyze significant differences between related data sets. For non‐normally distributed data, we applied the Wilcoxon signed‐rank tests, while for normally distributed data, we used *t*‐tests. The median and interquartile range (IQR) were used for skewed data, and the mean and standard deviation (SD) were used for normal data.

## 3 RESULTS

The results in Table 1 show that PDI_air_ has a higher γ passing rate, lower mean γ value, and lower percentage of *γ* > 1.5 than PDI_predicted_. This indicates that the EPID is accurately calibrated and that the difference between the measured and calculated values is minimal.

**TABLE 1:** γ analysis results of PDI_air,_ with PDI_predicted_ as the reference.^a^

γ criteria	γ passing rate (%)	Percentage of γ > 1.5 (%)	Mean γ value
3%/3 mm	99.9 (99.8, 100)	0.0 (0.0, 0.0)	0.20±0.03
3%/2 mm	99.6 (99.3, 99.9)	0.0 (0.0, 0.0)	0.26±0.06
2%/2 mm	98.2 (99.5, 96.6)	0.1 (0.0, 0.2)	0.30±0.05

^a^ The mean and SD were used to describe the distributions of normally distributed data, while the median and IQR were used for skewed data. PDI_air_: portal dose image measured without the phantom in air. PDI_predicted_: TPS predicted portal dose image without the phantom in air. The γ analysis results in the table represent the statistical findings for all fields across the four radiation treatment plans designed for this study.

Tables 2, 3, 4 present the *γ* passing rate, mean *γ* value, and percentage of *γ* > 1.5 for all groups of back‐projected 2D entrance fluences (vs. PDI_air_) at 2%/2 mm and the corresponding statistical results. The *γ* results at 3%/3 mm and 3%/2 mm are shown in Table S1. All three *γ* results were within the tolerance limits recommended by TG‐218 for BP_metal‐free_, demonstrating that the back‐projection algorithm used in this study is clinically acceptable for a metal‐free phantoms.^20^ However, compared to BP_metal‐free_, all three *γ* results were significantly worse (*p* < 0.01 or *p* < 0.001, Wilcoxon signed‐rank test) for the back‐projected 2D entrance fluences of the metal‐contacting phantom. BP_Ti artifact‐free_ had significantly lower mean γ values than BP_Ti_ (*p* < 0.05, Wilcoxon signed‐rank test, Table 4) for most beam fields (Figure S3). However, the other γ results did not show significant differences among BP_Ti_, BP_Ti artifact‐free_, BP_Al_, and BP_Al artifact‐free_ (*p* > 0.05, Wilcoxon signed‐rank test).

**TABLE 2:** γ passing rate for the back‐projected 2D entrance fluence versus PDI_air_, and the statistical results.

	γ passing rate versus PDI_air_ (%) ^a^, (2%/2 mm)	*P* values associated with the γ analysis results for different groups of data (Wilcoxon signed‐rank test)			
		vs. BP_Ti_	vs. BP_Ti artifact‐free_	vs. BP_Al_	vs. BP_Al artifact‐free_
BP_metal‐free_	92.4 (87.4, 95.4)	*p* =0.004**	*p* =0.001**	*p* =0.001**	*p* <0.001***
BP_Ti_	90.5 (87.2, 91.7)	–	*p* =0.232	*p* =0.399	*p* =0.925
BP_Ti artifact‐free_	90.7 (86.2, 93.0)	–	–	*p* =1.000	*p* =0.493
BP_Al_	90.6 (86.8, 93.9)	–	–	–	*p* =0.169
BP_Al artifact‐free_	91.0 (85.7, 93.8)	–	–	–	–

PDI_air_: portal dose image measured without the phantom in air. a: The distribution of the skewed data is presented using the median and IQR. BP_metal‐free_: 2D entrance fluences obtained using the back‐projection algorithm for a phantom without metal. BP_Ti_ and BP_Al_: 2D entrance fluences obtained using the back‐projection algorithm for phantoms containing titanium/aluminum rods; and the corresponding CT images contain metal artifacts. BP_Ti artifact‐free_ and BP_Al artifact‐free_: 2D entrance fluences obtained using the back‐projection algorithm for phantoms containing titanium/aluminum rods; the corresponding CT images do not contain metal artifacts. **: *p* value < 0.01. ***: *p* value < 0.001. The γ analysis results in the table represent the statistical findings for all fields across the four radiation treatment plans designed for this study.

**TABLE 3:** Percentage of γ>1.5 for the back‐projected 2D entrance fluences versus PDI_air_, and the statistical results.

	Percentage of γ>1.5 versus PDI_air_ (%) ^a^, (2%/2 mm)	*P* values associated with γ analysis results for different groups of data (Wilcoxon signed‐rank test)			
		vs. BP_Ti_	vs. BP_Ti artifact‐free_	vs. BP_Al_	vs. BP_Al artifact‐free_
BP_metal‐free_	2.2 (0.9, 3.0)	*p* < 0.001***	*p* <0.001***	*p* <0.001***	*p* <0.001***
BP_Ti_	3.1 (1.5, 4.7)	–	*p* =0.303	*p* =0.475	*p* =0.989
BP_Ti artifact‐free_	2.7 (1.8, 4.4)	–	–	*p* =0.927	*p* =0.455
BP_Al_	2.9 (1.5, 4.8)	–	–	–	*p* =0.208
BP_Al artifact‐free_	3.0 (1.8, 5.2)	–	–	–	–

PDI_air_: portal dose image without the phantom in air. a: The distribution of the skewed data is presented using the median and IQR. BP_metal‐free_: 2D entrance fluences obtained using the back‐projection algorithm for a phantom without metal. BP_Ti_ and BP_Al_: 2D entrance fluences obtained using the back‐projection algorithm for phantoms containing titanium/aluminum rods; the corresponding CT images contain metal artifacts. BP_Ti artifact‐free_ and BP_Al artifact‐free_: 2D entrance fluences obtained using the back‐projection algorithm for phantoms containing titanium/aluminum rods; the corresponding CT images do not contain metal artifacts. ***: *p* value<0.001. The γ analysis results in the table represent the statistical findings for all fields across the four radiation treatment plans designed for this study.

**TABLE 4:** Mean ***γ*** value for the back‐projected 2D entrance fluences versus PDI_air_, and the statistical results.

	Mean γ value versus PDI_air_^a^, (2%/2 mm)	P values associated with γ analysis results for different groups of data (Wilcoxon signed‐rank test)			
		vs. BP_Ti_	vs. BP_Ti artifact‐free_	vs. BP_Al_	vs. BP_Al artifact‐free_
BP_metal‐free_	0.38 (0.34, 0.46)	*p* =0.001**	*p* < 0.001***	*p* < 0.001***	*p* < 0.001***
BP_Ti_	0.42 (0.39, 0.48)	–	*p* < 0.05^*,b^	*p* =0.245	*p* =0.422
BP_Ti artifact‐free_	0.42 (0.37, 0.49)	–	–	*p* =0.722	*p* =0.636
BP_Al_	0.43 (0.37, 0.49)	–	–	–	*p* =0.521
BP_Al artifact‐free_	0.42 (0.37, 0.50)	–	–	–	–

PDI_air_: portal dose image without the phantom in air. a: The distribution of the skewed data is presented using the median and IQR. b: Supplement figure3 shows the difference between the mean γ values of BP_Ti_ and BP_Ti artifact‐free_ corresponding to each beam field, using PDI_air_ as a reference. BP_metal‐free_: 2D entrance fluences obtained using the back‐projection algorithm for a phantom without metal. BP_Ti_ and BP_Al_: 2D entrance fluences obtained using the back‐projection algorithm for phantoms containing titanium/aluminum rods; the corresponding CT images contain metal artifacts. BP_Ti artifact‐free_ and BP_Al artifact‐free_: 2D entrance fluences obtained using the back‐projection algorithm for phantoms containing titanium/aluminum rods; the corresponding CT images do not contain metal artifacts. *: p value<0.05. **: *p* value < 0.01. ***: *p* value < 0.001. The γ analysis results in the table represent the statistical findings for all fields across the four radiation treatment plans designed for this study.

The accuracy of the back‐projected 2D entrance fluence for the metal‐containing phantom depends on several factors, such as the CT image quality, mechanical errors, absolute dose errors, positional errors, and errors in the back‐projection algorithm. Therefore, using PDI_air_ as a reference for γ analysis does not adequately reflect the effects of different metals and metal artifacts on the back‐projection algorithm. To isolate the effects of metal and metal artifacts from other factors, we used BP_metal‐free_, which corresponds to the metal‐free phantom, as the reference for γ analysis. Tables 5, 6, 7 show that there was no significant difference (*p* > 0.05, Wilcoxon signed‐rank test) between BP_Al_ and BP_Al artifact‐free_ for all three γ criteria, indicating that aluminum‐induced metal artifacts had negligible effects on the accuracy of the back‐projected 2D entrance fluence. In contrast, BP_Ti artifact‐free_ had significantly better γ results than BP_Ti_ for all three criteria (*p* < 0.001, Wilcoxon signed‐rank test), indicating that titanium‐induced metal artifacts had substantial impacts on the accuracy of the back‐projected 2D entrance fluence and that removing these artifacts considerably improved the accuracy. In addition, BP_Ti_ had significantly worse γ results than BP_Al_ and BP_Al artifact‐free_ for all three criteria (*p* < 0.001, Wilcoxon signed‐rank test), while BP_Ti artifact‐free_ did not differ significantly from BP_Al_ and BP_Al artifact‐free_ in terms of the mean γ value (*p* > 0.05, paired t test). This implies that titanium and its metal artifacts in CT images introduce more errors in the back‐projected 2D entrance fluence than aluminum and that eliminating these artifacts reduces the error, leading to values more consistent with the aluminum‐containing phantom (mean γ value).

**TABLE 5:** γ passing rate for the back‐projected 2D entrance fluences of metal‐containing phantoms versus BP_metal‐free_, and the statistical results.

	γ passing rate versus BP_metal‐free_ (%) ^a^, (2%/2 mm)	*p* values associated with γ analysis results for different groups of data (Wilcoxon signed‐rank test)			
		vs. BP_Ti_	vs. BP_Ti artifact‐free_	vs. BP_Al_	vs. BP_Al artifact‐free_
BP_Ti_	99.2 (98.9, 99.8)	–	*p* < 0.001***	*p* < 0.001***	*p* < 0.001***
BP_Ti artifact‐free_	99.7 (99.2, 100.0)	–	–	*p* =0.005**	*p* =0.008**
BP_Al_	99.9 (99.8, 100.0)	–	–	–	*p* =0.329
BP_Al artifact‐free_	99.9 (99.7, 100.0)	–	–	–	–

^a^: The distribution of the skewed data is presented using the median and IQR. BP_metal‐free_: 2D entrance fluences obtained using the back‐projection algorithm for a phantom without metal. BP_Ti_ and BP_Al_: 2D entrance fluences obtained using the back‐projection algorithm for phantoms containing titanium/aluminum rods and the corresponding CT images contain metal artifacts. BP_Ti artifact‐free_ and BP_Al artifact‐free_: 2D entrance fluences obtained using the back‐projection algorithm for phantoms containing titanium/aluminum rods and the corresponding CT images do not contain metal artifacts. **: *p* value<0.01. ***: *p* value<0.001. The γ analysis results in the table represent the statistical findings for all fields across the four radiation treatment plans designed for this study.

**TABLE 6:** Percentage of γ>1.5 for the back‐projected 2D entrance fluences of metal‐containing phantoms versus BP_metal‐free_, and the statistical results.

	Percentage of γ>1.5 versus BP_metal‐free_ (%) ^a^, (2%/2 mm)	P values associated with γ analysis results for different groups of data (Wilcoxon signed‐rank test)			
		vs. BP_Ti_	vs. BP_Ti artifact‐free_	vs. BP_Al_	vs. BP_Al artifact‐free_
BP_Ti_	0.2 (0.0, 0.2)	–	*p* < 0.001***	*p* < 0.001***	*p* < 0.001***
BP_Ti artifact‐free_	0.0 (0.0, 0.1)	–	–	*p* =0.011*	*p* =0.016*
BP_Al_	0.0 (0.0, 0.0)	–	–	–	*p* =0.317
BP_Al artifact‐free_	0.0 (0.0, 0.0)	–	–	–	–

^a^The distribution of the skewed data is presented using the median and IQR. BP_metal‐free_: 2D entrance fluences obtained using the back‐projection algorithm for a phantom without metal. BP_Ti_ nd BP_Al_: 2D entrance fluences obtained using the back‐projection algorithm for phantoms containing titanium/aluminum rods; the corresponding CT images contain metal artifacts. BP_Ti artifact‐free_ and BP_Al artifact‐free_: 2D entrance fluences obtained using the back‐projection algorithm for phantoms containing titanium/aluminum rods and the corresponding CT images do not contain metal artifacts. *: *p* value< 0.05. ***: *p* value<0.001. The γ analysis results in the table represent the statistical findings for all fields across the four radiation treatment plans designed for this study.

**TABLE 7:** Mean γ value for the back‐projected 2D entrance fluences of metal‐containing phantoms versus BP_metal‐free_, and the statistical results.

	Mean γ value versus BP_metal‐free_^a^, (2%/2 mm)	*p* values associated with γ analysis results for different groups of data (Wilcoxon signed‐rank test or paired t test)			
		vs. BP_Ti_	vs. BP_Ti artifact‐free_	vs. BP_Al_	vs. BP_Al artifact‐free_
BP_Ti_	0.17±0.04 0.17 (0.13, 0.21)	–	*p* < 0.001*** ^b^	*p* =0.001** ^b^	*p* <0.001*** ^b^
BP_Ti artifact‐free_	0.15±0.04	–	–	*p* =0.524 ^c^	*p* =0.751 ^c^
BP_Al_	0.15±0.04	–	–	–	*p* =0.294 ^c^
BP_Al artifact‐free_	0.15±0.04	–	–	–	–

^a^The mean and SD are used to describe the distribution of normally distributed data, while the median and IQR are used for skewed data. In the case of BP_Ti_, despite the skewness of the data, the contrast was reported using the mean and SD.

^b^Wilcoxon signed‐rank test.

^c^Paired *t* test. BP_metal‐free_: 2D entrance fluences obtained using the back‐projection algorithm for a phantom without metal. BP_Ti_ and BP_Al_: 2D entrance fluences obtained using the back‐projection algorithm for phantoms containing titanium/aluminum rods; the corresponding CT images contain metal artifacts. BP_Ti artifact‐free_ and BP_Al artifact‐free_: 2D entrance fluences obtained using the back‐projection algorithm for phantoms containing titanium/aluminum rods and the corresponding CT images do not contain metal artifacts. **: *p* value<0.01. ***: *p* value<0.001. The γ analysis results in the table represent the statistical findings for all fields across the four radiation treatment plans designed for this study.

**FIGURE 2:**
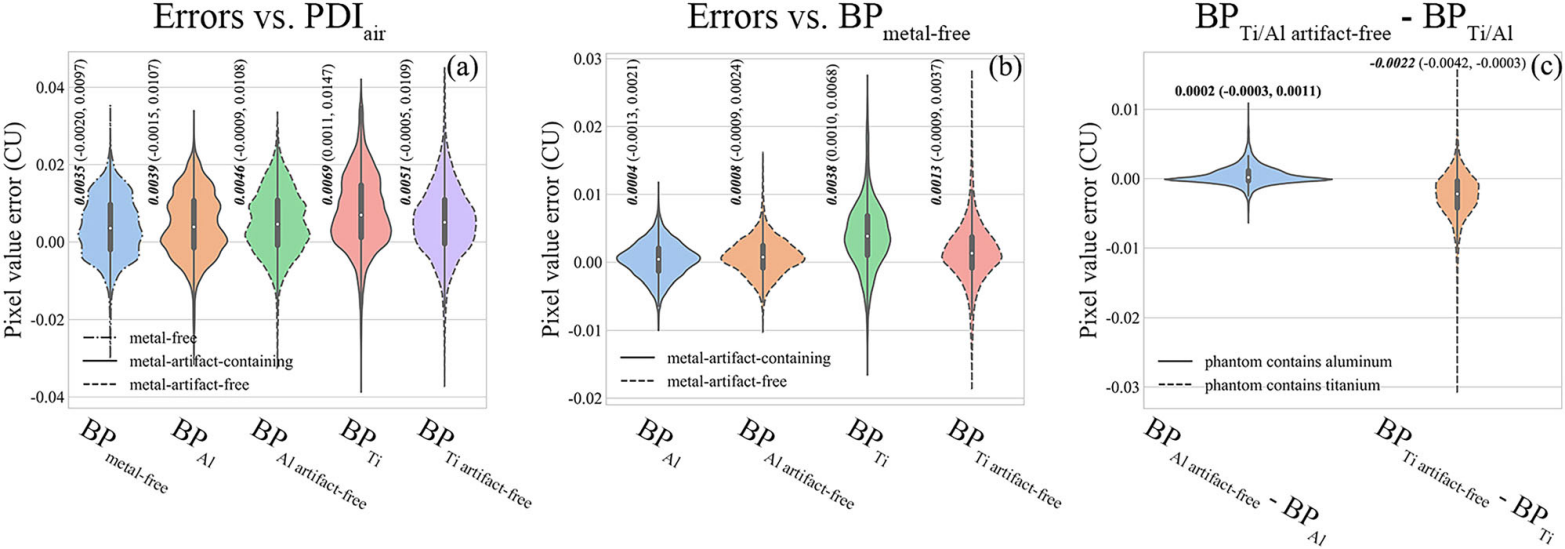
Violin plots of the pixel‐level differences in the metal‐affected regions for (a) all back‐projected 2D entrance fluences vs. PDI_air_, (b) metal‐containing back‐projected 2D entrance fluences (BP_Al_/BP_Al artifact‐free_ /BP_Ti_/BP_Ti artifact‐free_) vs. BP_metal‐free_ and (c) BP_Ti artifact‐free_/BP_Al artifact‐free_ vs. BP_Ti_/BP_Al_. The median and IQR of all pixel value errors are shown in the figure.

In the γ analysis, the accuracy of the 2D entrance fluence corresponding to the beam field is determined for the entire image. However, focusing on the metal‐affected regions allows us to better identify errors caused by metal and metal artifacts. Figure 2 compares the pixel‐level differences in the back‐projected 2D entrance fluence in the metal‐affected region with respect to different reference images. In Figure 2a, PDI_air_ is used as the reference, and the results show that BP_metal‐free_ has the smallest difference (median 0.0035 CU) and the narrowest distribution. BP_Al_ and BP_Al artifact‐free_ have slightly larger differences but similar distributions to BP_metal‐free_. BP_Ti_ has the largest difference (median 0.0069 CU) and the widest distribution, which can be reduced by considering BP_Ti artifact‐free_ (median 0.0051 CU). In Figure 2b, BP_metal‐free_ is used as the reference, and the results show that BP_Al_ and BP_Al artifact‐free_ have negligible differences (median 0.0004 and 0.0008 CU, respectively) and narrow distributions in the metal‐affected region. BP_Ti_ has a larger difference (median 0.0038 CU) and a wider distribution, which can also be reduced by BP_Ti artifact‐free_ (median 0.0013 CU). In Figure 2c, BP_Al_ and BP_Ti_ are used as references for their corresponding artifact‐free images, and the results show that BP_Al artifact‐free_ images have approximately no difference (median 0.0002 CU) and a very narrow distributions in the metal‐affected regions, while BP_Ti artifact‐free_ has a negative difference (median −0.0022 CU) and a wider distribution, indicating a greater reduction in pixel values due to the removal of Ti‐induced metal artifacts.

The pixel‐level MAE and RMSE of the back‐projected 2D entrance fluences for each beam field versus PDI_air_ in the metal‐affected regions are presented in Table 8. The MAE and RMSE values for BP_Al_ and BP_Al artifact‐free_ were slightly higher than those for BP_metal‐free_, whereas the MAE and RMSE values for BP_Ti_ and BP_Ti artifact‐free_ were considerably increased. The errors for BP_Al_ and BP_Al artifact‐free_ were comparable, while BP_Ti artifact‐free_ showed substantially lower MAE and RMSE values than BP_Ti_. When BP_metal‐free_ was used as the reference, the error comparison among BP_Al_, BP_Al artifact‐free_, BP_Ti_ and BP_Ti artifact‐free_ showed similar trends (Table 9). Compared to the MAE and RMSE values for BP_Ti_, the MAE and RMSE values for BP_Ti artifact‐free_ decreased from 0.0050 CU to 0.0034 CU and from 0.0063 CU to 0.0040 CU, respectively, compared to BP_Ti_.

**TABLE 8:** MAE and RMSE values of the back‐projected 2D entrance fluences versus PDI_air_ in the metal‐affected regions.

	MAE (vs. PDI_air_) (CU)	RMSE (vs. PDI_air_) (CU)
BP_metal‐free_	0.0070	0.0087
BP_Al_	0.0075	0.0094
BP_Al artifact‐free_	0.0077	0.0097
BP_Ti_	0.0098	0.0125
BP_Ti artifact‐free_	0.0082	0.0105

PDI_air_: portal dose image measured without the phantom in air. BP_metal‐free_: 2D entrance fluences obtained using the back‐projection algorithm for a phantom without metal. BP_Ti_ and BP_Al_: 2D entrance fluences obtained using the back‐projection algorithm for phantoms containing titanium/aluminum rods; the corresponding CT images contain metal artifacts. BP_Ti artifact‐free_ and BP_Al artifact‐free_: 2D entrance fluences obtained using the back‐projection algorithm for phantoms containing titanium/aluminum rods; the corresponding CT images do not contain metal artifacts. MAE: mean absolute error. RMSE: root mean square error. The table data represent the errors of metal‐affected pixel points in the 2D entrance fluences of all of the fields in this study as compared to the corresponding pixel points in the PDI_air_.

**TABLE 9:** MAE and RMSE values for the back‐projected 2D entrance fluences of metal‐containing vs. metal‐free phantoms in metal‐affected regions.

	MAE (vs. BP_metal‐free_) (CU)	RMSE (vs. BP_metal‐free_) (CU)
BP_Al_	0.0021	0.0027
BP_Al artifact‐free_	0.0022	0.0028
BP_Ti_	0.0050	0.0063
BP_Ti artifact‐free_	0.0034	0.0040

BP_metal‐free_: 2D entrance fluences obtained using the back‐projection algorithm for a phantom without metal. BP_Ti_ and BP_Al_: 2D entrance fluences obtained using the back‐projection algorithm for phantoms containing titanium/aluminum rods; the corresponding CT images contain metal artifacts. BP_Ti artifact‐free_ and BP_Al artifact‐free_: 2D entrance fluences obtained using the back‐projection algorithm for phantoms containing titanium/aluminum rods; the corresponding CT images do not contain metal artifacts. MAE: mean absolute error. RMSE: root mean square error. The table data represent the errors of metal‐affected pixel points in the 2D entrance fluences of all of the fields in this study for metal‐containing phantoms as compared to the corresponding pixel points in the BP_metal‐free_.

**FIGURE 3:**
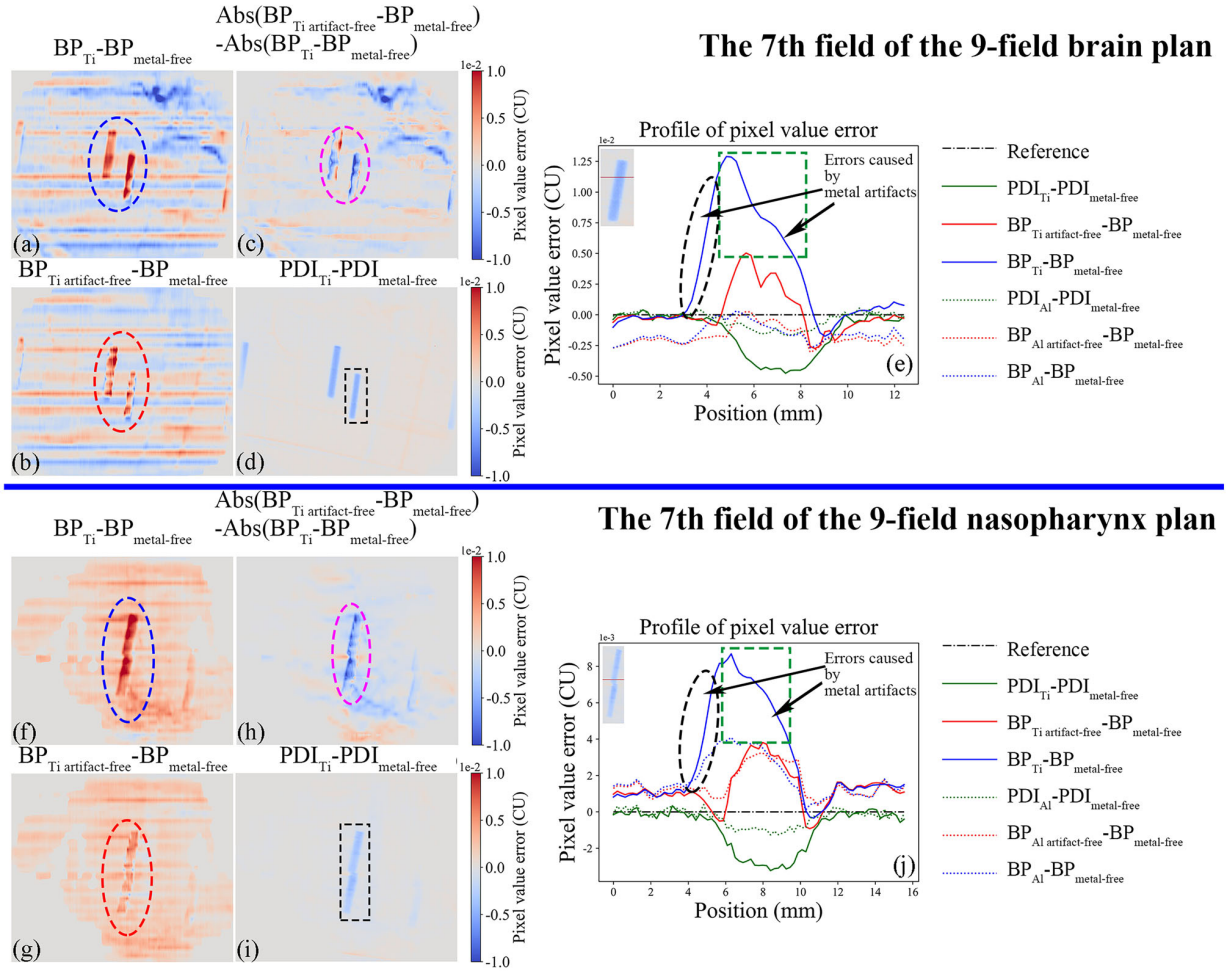
(a) and (f) Dose difference maps between BP_Ti_ and BP_metal‐free_. (b) and (g) Dose difference maps between BP_Ti artifact‐free_ and BP_metal‐free_. (c) and (h) Difference maps of absolute dose errors of BP_Ti_ and BP_Ti artifact‐free_ relative to BP_metal‐free_. In (a), (b), (f), and (g), the darker red color indicates larger errors in the back‐projected 2D entrance fluence for the Ti‐containing phantom than for the metal‐free phantom, while the darker blue color indicates the opposite. In (c) and (h), the darker blue color indicates smaller absolute errors in BP_Ti artifact‐free_ than in BP_Ti_, using the BP_metal‐free_ as a reference, while the darker red color indicates the opposite. (d) and (i) Dose difference maps of PDI_Ti_ and PDI_metal‐free_. The blue area in the images represents the metal region. (e) and (j) Error value profiles of BP_Ti_/ BP_Ti artifact‐free_/ BP_Al_/ BP_Al artifact‐free_ relative to BP_metal‐free_, and pixel value difference profiles of PDI_Ti_/ PDI_Al_ relative to PDI_metal‐free_. These curves correspond to the red lines passing through the metal region in the small image in the upper left corner. The error in the metal region in BP_Ti_ is larger than that in BP_Ti artifact‐free_. The black arrows in (e) and (j) indicate the error caused by metal artifacts in BP_Ti_.

Figure 3 illustrates the effect of metal and metal artifacts on the back‐projected 2D entrance fluences for field seven in the nine‐field plans for the brain and nasopharynx cases, respectively. The back‐projected 2D entrance fluences for other beam fields are shown in Figures S4–S7. Metal inserts attenuate and scatter more X‐rays, resulting in lower PDI pixel values in the metal‐affected region than in the PDI_metal‐free_ (blue regions in Figure 3d and i). In the brain case (upper part of Figure 3), the metal‐affected region (elliptical dashed line) has a darker red color in Figures 3a and b, indicating larger discrepancies between BP_Ti_ and BP_metal‐free_, and between BP_Ti artifact‐free_ and BP_metal‐free_, respectively. The lighter red color in Figure 3b than in Figure 3a suggests that correcting the metal artifacts in the CT images can reduce the errors in the back‐projected 2D entrance fluence in the metal‐affected regions. The predominantly blue color in Figure 3c (pink dashed ellipse) shows that BP_Ti artifact‐free_ has a much smaller absolute error in the metal‐affected region than BP_Ti_. The profile of pixel error values in Figure 3e further confirms that BP_Ti_ has higher error values and a wider distribution of error pixels in the metal‐affected region than BP_Ti artifact‐free_ (green dashed box and black dashed ellipse). For the aluminum case, there is no substantial difference between BP_Al artifact‐free_ and BP_Al_ in the metal‐affected region, and both have smaller errors than BP_Ti artifact‐free_ and BP_Ti_ . A similar pattern is observed for the nasopharyngeal case (lower part of Figure 3).

**FIGURE 4:**
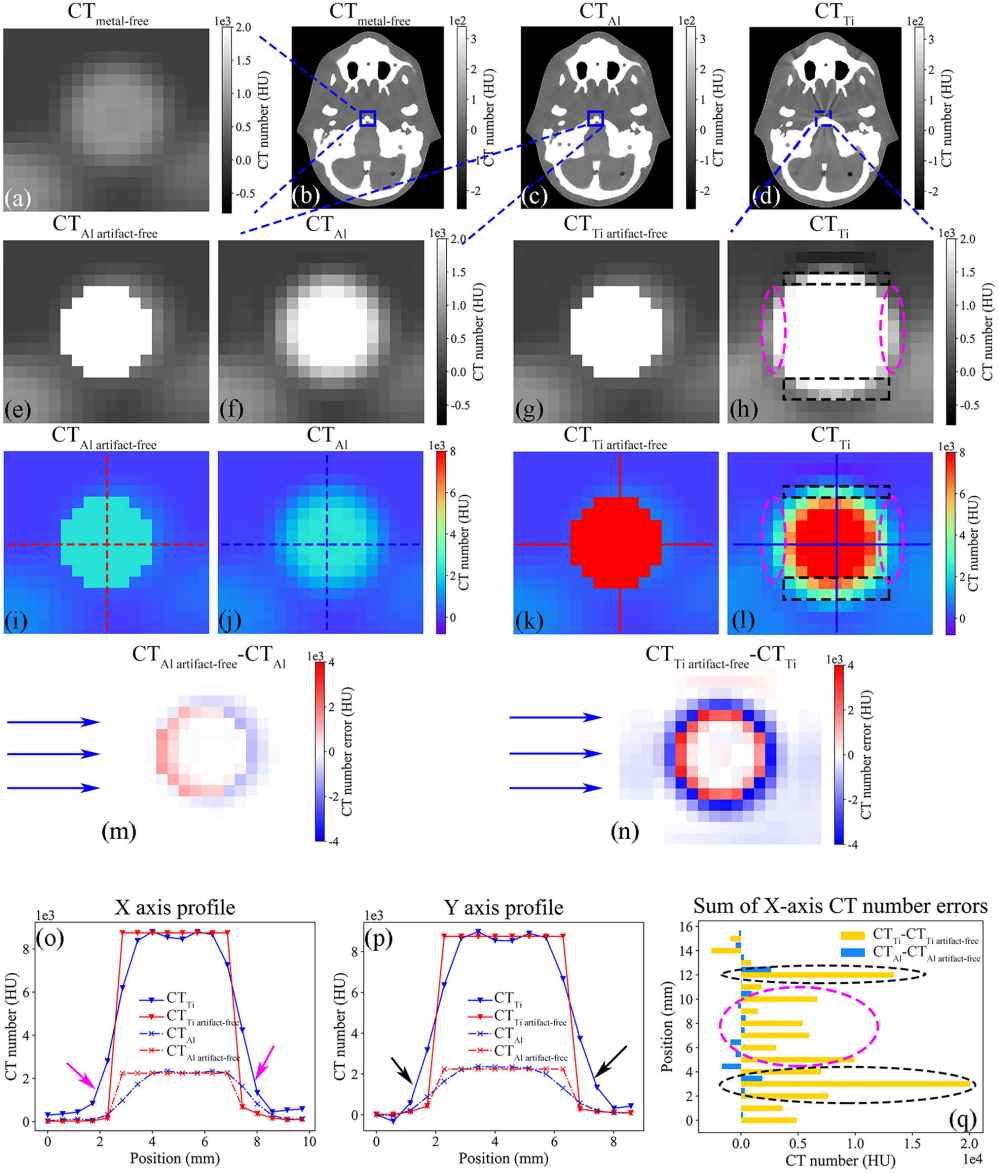
Schematic diagram illustrating the increased errors in the metal region in BP_Ti_ caused by metal artifacts. The original CT images of the metal‐free phantom (CT_metal‐free_), Al‐containing phantom (CT_Al_), and Ti‐containing phantom (CT_Ti_) are displayed in (b)–(d), with a display window of [−260, 340] HU. Magnified images of the metal regions in the blue boxes in (b)‐(d) are shown in (a), (f), and (h). (e) CT image of the Al‐containing phantom without metal artifacts (CT_Al artifact‐free_), corresponding to (f). (g) CT image of the Ti‐containing phantom without metal artifacts (CT_Ti artifact‐free_), corresponding to (h). Color images corresponding to (e)–(h) are shown in (i)–(l), with a display window of [−800, 2000] HU. (m) Difference distribution map between CT_Al artifact‐free_ and CT_Al_. (n) Difference distribution map between CT_Ti artifact‐free_ and CT_Ti_, with a display window of [−4000, 4000] HU. The CT value profiles of the horizontal and vertical lines passing through the voxels in (i)–(l) are presented in (o) and (p), respectively. The blue arrows in (m) and (n) represent the X‐ray transmission direction. (q) Sum of CT value errors between CT_Ti artifact‐free_ and CT_Ti_ in (n) and between CT_Al artifact‐free_ and CT_Al_ in (m) along the X‐ray transmission direction. Metal artifacts due to Al were less severe than those due to Ti, and the differences in the CT values due to the metal artifacts in CT_Al_ and CT_Al artifact‐free_ were smaller than those in CT_Ti_ and CT_Ti artifact‐free_.

The mechanism of how metal artifacts affect the back projection algorithm (Figure 4) was investigated by comparing the CT value distribution around a metal insert before and after MAR. The case with the aluminum insert had a lower RED value (Figure 4f, j, o and p) and caused weak metal artifacts in CT_Al_ (Figure 4c, m, n and p), resulting in a small difference in the sum of the CT values between CT_Al_ and CT_Al artifact‐free_ (185 ± 1014 HU for CT_Al_ −CT_Al artifact‐free_ along the x‐axis direction in Figure 4q). The case with the titanium insert had a higher RED value (Figure 4 h, l, o and p) and caused severe metal artifacts in CT_Ti_ (Figure 4d and n–p), leading to a large difference in the sum of the CT values between CT_Ti_ and CT_Ti artifact‐free_ (5518±5600 HU for CT_Ti_ −CT_Ti artifact‐free_ along the *x*‐axis direction in Figure 4q).

## 4 DISCUSSION

An EPID records the spatial distribution of the X‐ray fluence exiting the patient during radiotherapy, and a back‐projection algorithm can be applied to the EPID image to reconstruct the 2D entrance fluence at the patient's skin surface based on the patient's CT images. However, the presence of metal implants in the body can lead to numerous metal artifacts in the CT images, affecting the accuracy of the CT values and ultimately the accuracy of the back‐projection result. Despite this issue, there are no existing studies on this topic. Therefore, in this paper, we report the errors caused by metal artifacts in CT images on back‐projected 2D entrance fluences and compare the effects of different metals on these errors. We created metal‐free, Ti‐containing, and Al‐containing phantoms by replacing the cylindrical inserts in the simulated human phantom. The planning CT images of the metal‐containing phantoms had metal artifacts. For comparison, we generated synthetic CT images with metal but without metal artifacts by replacing the voxel values of the non‐metal inserts in the metal‐free phantom with those of Ti or Al. We used the planning CT images of the phantom to obtain the 2D entrance fluences in the back‐projection calculation, assuming that the setup error was negligible (<1 mm in all directions) due to the rigid structure and easy positioning of the phantom. We acquired PDIs corresponding to the metal‐free and metal‐containing phantoms using the EPID system and obtained different 2D entrance fluences based on the respective CT images using commercial software (KylinRay‐Dose4D). We evaluated the error of the back‐projection algorithm caused by the metal and metal artifacts and the improvement in the accuracy after removing metal artifacts from the CT images of the back‐projected 2D entrance fluence by comparing the γ results and the pixel value error in the metal‐affected region.

According to TG‐218, the universal tolerance limit for the *γ* passing rate is 95% with 3%/2 mm and a 10% dose threshold.^20^ The *γ* results of BP_metal‐free_ and PDI_air_ in this study indicate that the back‐projection algorithm we used is accurate when the phantom does not contain any metals. In a study by Wendling et al., a back‐projection technique was developed that achieved *γ* passing rates (2%/2 mm) of 99.86%–99.99% between the 2D PDIs and the measured dose distribution of the film for a homogeneous slab phantom and IMRT plan, with a mean γ value of less than 0.4.^22^ For the improved back‐projection algorithm for non‐uniform media in patients developed by Wendling et al., the average γ passing rate and mean γ value of the PDIs relative to the TPS calculations were 93.1% and 0.40, respectively, with 3%/3 mm and a 20% dose threshold.^23^ Our models demonstrate relatively competitive accuracy.

When the phantom contains metals with high RED values, such as titanium, removing metal artifacts from CT images can improve the accuracy of 2D entrance fluences in metal‐affected regions obtained by back‐projection algorithms. The 2D entrance fluence is reconstructed by performing a back‐projection calculation using the primary beam distribution measured by the EPID system. This calculation accounts for the X‐ray attenuation along the propagation path through the patient or phantom, which is determined by the RED distribution derived based on the CT values in the CT images. However, metal artifacts can distort the CT values distribution in the CT images, resulting in errors in the 2D entrance fluence in the metal‐affected regions. Titanium produced many obvious metal artifacts in the CT images (Figure 4d), and the bright artifacts in CT_Ti_ on both sides of the propagated beam (black ellipses in Figure 4h and l, black arrows in Figure 4p) led to high CT values in regions outside the geometric contours of the metal on both sides of the beam propagation direction (black dashed line in Figure 4q), resulting in errors in a larger range of pixels in the metal‐affected region of the BP_Ti_ than in the BP_Ti artifact‐free_ (black ellipses in Figure 3e and j). In addition, the bright artifacts in the metal‐affected regions (pink ellipses in Figure 4h and l and pink arrows in Figure 4o) increase the sum of the CT values along the x‐ray path (pink dashed ellipses in Figure 4q), resulting in much larger error values in the metal‐affected region in BP_Ti_ than in BP_Ti artifact‐free_ (green rectangular boxes in Figure 3e and j). Therefore, removing metal artifacts caused by titanium in CT images improves the accuracy of CT values in metal‐affected regions, which substantially reduces the error in the metal‐affected regions in the 2D entrance fluences obtained by the back‐projection algorithm (Figure 2, Tables 8, 9) and significantly improves the γ results (*p* < 0.05, Tables 5, 6, 7). In contrast, the RED value of aluminum is low, and aluminum produces insignificant metal artifacts in the CT images (Figure 4c). Moreover, because the difference in the CT values in the metal‐affected regions between CT_Al_ and CT_Al artifact‐free_ is not substantial (Figure 4q), the difference in the pixel values in the metal‐affected region between BP_Al artifact‐free_ and BP_Al_ is small (Figure 2 and Tables 8, 9), and their γ results are similar (*p* > 0.05, Tables 5, 6, 7).

The pixel values in the metal‐affected regions of the CT images should be similar between BP_Ti artifact‐free_/BP_Al artifact‐free_ and BP_metal‐free_ after MAR; however, this was not observed in our results. BP_Ti artifact‐free_ and BP_Al artifact‐free_ showed worse γ results than BP_metal‐free_ (vs. PDI_air_, *p* < 0.05, Tables 2, 3, 4), and substantial pixel value discrepancies were observed in the metal‐affected region (Figures 2, 3, Table 9). The errors in BP_Ti artifact‐free_ were larger than those in BP_Al artifact‐free_. The increased photon scattering caused by the higher RED values of metals compared to those of normal tissue may affect the accuracy of the back‐projection algorithm, and this effect may be more pronounced for metals with higher RED values. This hypothesis requires further validation in future studies.

Our previous study investigated the effect of an MAR technique on dose calculation errors for PTVs and OARs in CT images of head tumor patients with metal dentures. We found that the MAR technique significantly changed the mean dose to the PTV and OARs, and the absolute differences for the PTV, oral mucosa, mandible, and parotid glands were 22.1 ± 17.9 cGy, 36.4±26.1 cGy, 32.0± 26.3 cGy, and 14.7 ±11.3 cGy, respectively (*p* <0.05), when the prescribed dose to the PTV was 6000 cGy.^15^ Metal artifacts in the CT images introduce errors in both the back projection of the 2D entrance fluence and the forward calculation of the dose. These errors are propagated and amplified in the EPID‐based in vivo 3D dose distribution, and this issue should be investigated in a future study.

In conclusion, this study showed that MAR for high RED value implants, such as titanium, can substantially reduce errors in back‐projected 2D entrance fluences. This can improve the accuracy of in vivo dose reconstructions near metal implants based on EPID measurements for patients receiving adaptive radiotherapy.

## 5 CONCLUSION

The purpose of this study was to investigate the effect of metal artifact reduction on the accuracy of 2D entrance fluences derived from EPID images using a back‐projection algorithm. For the phantom without metal, the γ passing rate of the back‐projected 2D entrance fluence relative to the PDI_air_ was 92.4% (2%/2 mm), which met the clinical acceptance criteria. However, for the phantoms with titanium and aluminum, the γ passing rates decreased to 90.5% (BP_Ti_) and 90.6% (BP_Al_), respectively. Aluminum caused less severe metal artifacts in the CT images than titanium, and MAR did not significantly improve the accuracy of the back‐projected 2D entrance fluence. In contrast, titanium caused more severe metal artifacts in the CT images, and MAR substantially reduced the pixel values of the back‐projected 2D entrance fluences in the metal‐affected regions (median 0.0022 CU) and improved the accuracy (MAE (vs. BP_metal‐free_) decreased from 0.0050 CU to 0.0034 CU, and RMSE (vs. BP_metal‐free_) decreased from 0.0063 CU to 0.0040 CU). In addition, MAR improved the γ evaluation results of BP_Ti artifact‐free_ (2%/2 mm, vs. BP_metal‐free_) compared to BP_Ti_ (γ passing rate increased from 99.2% to 99.7%, percentage of γ> 1.5 decreased from 0.2% to 0%, and mean γ value decreased from 0.17 to 0.15). These results indicate that for patients with high RED value metallic implants (e.g., titanium) in the body, MAR based on their CT images is essential to enhance the accuracy of the back‐projected 2D entrance fluence obtained from EPID images in adaptive radiotherapy. This enhanced accuracy will improve the accuracy of in vivo 3D dose distributions, allowing more accurate assessments of patient target volume doses and radiation damage, which has important clinical implications.

## AUTHOR CONTRIBUTIONS

Zheng Cao conceived the experiments. Xiang Gao acquired and analyzed the data for the work. Zheng Cao, Xiang Gao, Gongfa Liu, and Yuanji Pei designed the study and analyzed the result. Zheng Cao, Xiang Gao, Gongfa Liu, and Yuanji Pei participated in writing manuscript. The final version of the manuscript has been reviewed and approved for publication by all authors.

## CONFLICT OF INTEREST STATEMENT

The authors declare no conflict of interest.

## Supporting information

Supporting Information

Supporting Information

Supporting Information

Supporting Information

Supporting Information

Supporting Information

Supporting Information

Supporting Information

## Data Availability

The original contributions presented in the study are included in the article. Further inquiries can be directed to the corresponding authors.
