# Supplementary material for: Effect of metal implants and metal artifacts on back‐projected two‐dimensional entrance fluence determined by EPID dosimetry
Source: J Appl Clin Med Phys. 2023 Aug 13;24(11):e14115. doi: 10.1002/acm2.14115 (PMC10647983; doi:10.1002/acm2.14115)
Supplement: Supplementary file 1 — Supporting Information [file ACM2-24-e14115-s005.docx]

Table S1 $\gamma$ analysis results for the back-projected 2D entrance fluence versus PDI_air_

|  | $\gamma$ passing rate versus PDI_air_ (%), (median, IQR) | |
| --- | --- | --- |
| $\gamma$ criteria | 3%/3 mm | 3%/2 mm |
| BP_metal-free_ | 97.9 (97.0, 99.1) | 95.8 (94.6, 98.0) |
| BP_Ti_ | 97.0 (95.3, 98.5) | 94.6 (92.3, 97.4) |
| BP_Ti artifact-free_ | 97.3 (95.6, 98.2) | 95.1 (93.1, 97.2) |
| BP_Al_ | 97.2 (95.3, 98.5) | 95.5 (91.9, 97.1) |
| BP_Al artifact-free_ | 97.0 (94.8, 98.2) | 95.3 (91.5, 96.9) |
|  | Percentage of $\gamma>$1.5 versus PDI_air_ (%), (median, IQR) | |
| $\gamma$ criteria | 3%/3 mm | 3%/2 mm |
| BP_metal-free_ | 0.2 (0.0, 0.4) | 0.3 (0.0, 1.1) |
| BP_Ti_ | 0.5 (0.1, 1.6) | 1.1 (0.4, 2.5) |
| BP_Ti artifact-free_ | 0.5 (0.1, 1.7) | 1.0 (0.3, 2.7) |
| BP_Al_ | 0.5 (0.1, 1.6) | 0.9 (0.3, 2.7) |
| BP_Al artifact-free_ | 0.6 (0.1, 1.7) | 0.9 (0.4, 2.8) |
|  | Mean $\gamma$ value versus PDI_air_, (median, IQR) | |
| $\gamma$ criteria | 3%/3 mm | 3%/2 mm |
| BP_metal-free_ | 0.25 (0.23, 0.31) | 0.33 (0.28, 0.37) |
| BP_Ti_ | 0.28 (0.26, 0.32) | 0.36 (0.32, 0.39) |
| BP_Ti artifact-free_ | 0.28 (0.24, 0.33) | 0.34 (0.32, 0.38) |
| BP_Al_ | 0.29 (0.25, 0.33) | 0.35 (0.30, 0.42) |
| BP_Al artifact-free_ | 0.28 (0.25, 0.33) | 0.35 (0.31, 0.42) |

PDI_air_: portal dose image without the phantom in air. BP_metal-free_: 2D entrance fluences obtained using the back-projection algorithm for a phantom without metal. BP_Ti_ and BP_Al_: 2D entrance fluences obtained using the back-projection algorithm for phantoms containing titanium/aluminum rods; the corresponding CT images contain metal artifacts. BP_Ti artifact-free_ and BP_Al artifact-free_: 2D entrance fluences obtained using the back-projection algorithm for phantoms containing titanium/aluminum rods; the corresponding CT images do not contain metal artifacts.
