# Supplementary figures and images for: Effect of metal implants and metal artifacts on back‐projected two‐dimensional entrance fluence determined by EPID dosimetry
Source: J Appl Clin Med Phys. 2023 Aug 13;24(11):e14115. doi: 10.1002/acm2.14115 (PMC10647983; doi:10.1002/acm2.14115)

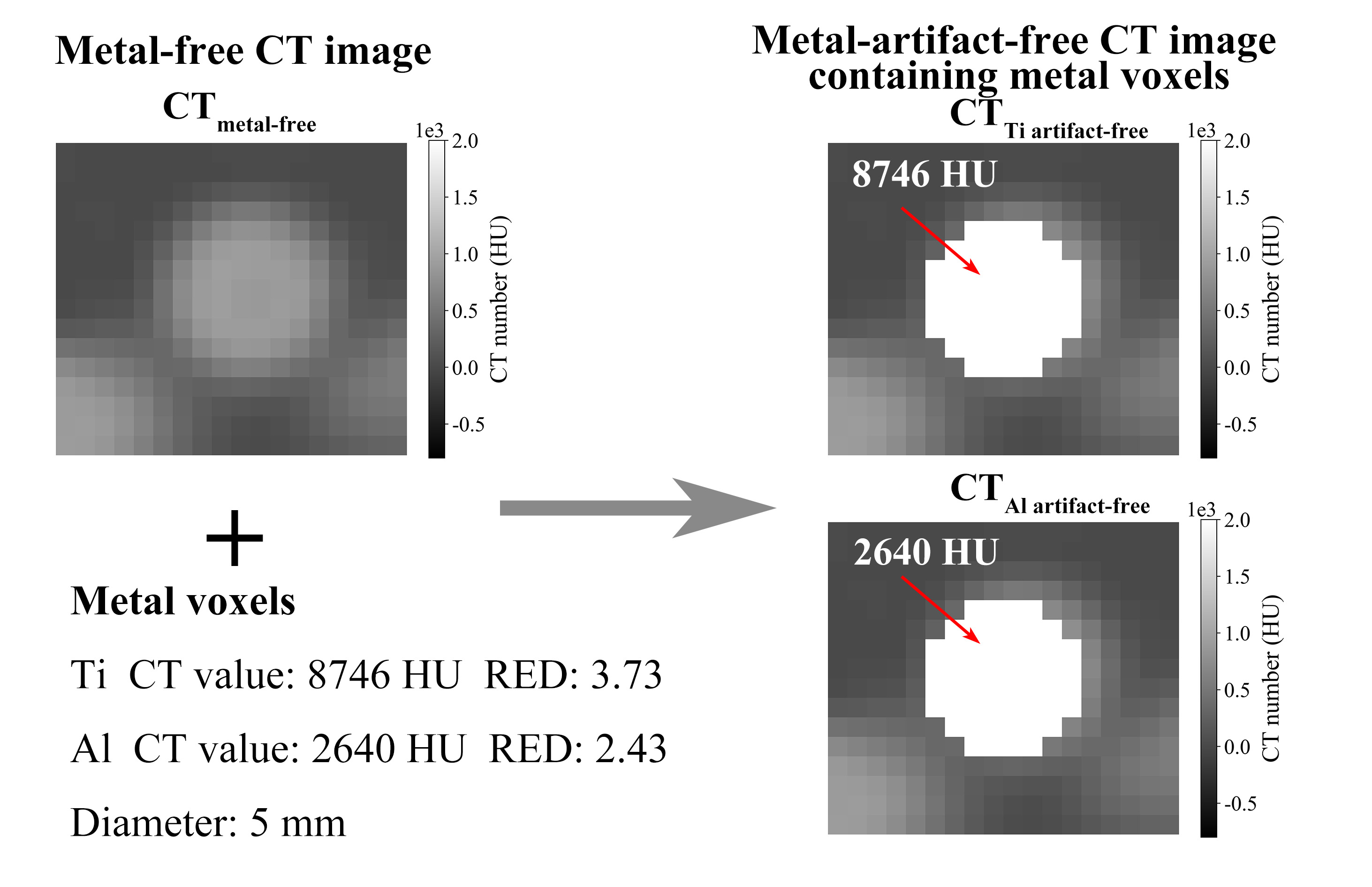

Supplement: Supplementary file 2 — Supporting Information [file ACM2-24-e14115-s007.jpg]

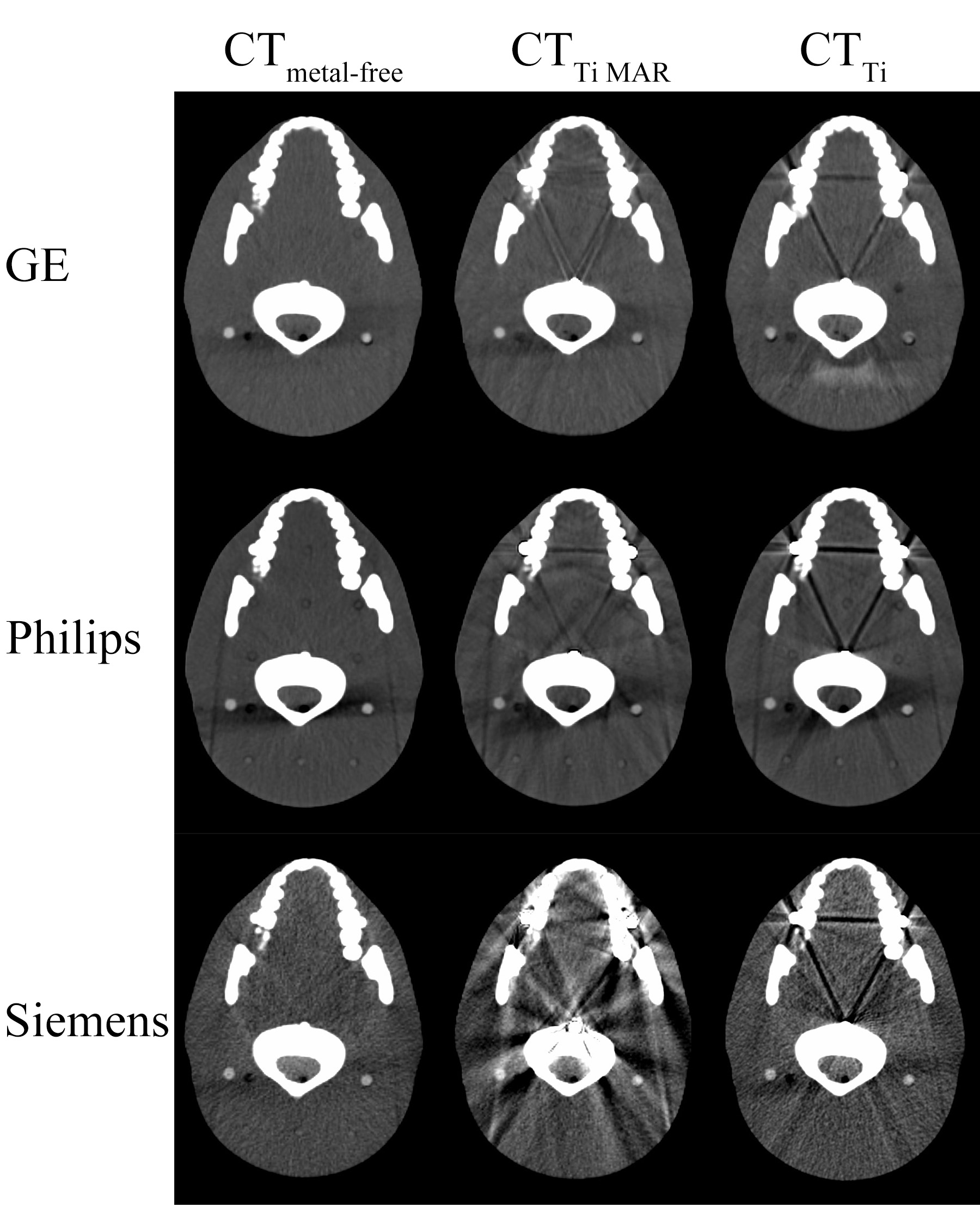

Supplement: Supplementary file 3 — Supporting Information [file ACM2-24-e14115-s004.jpg]

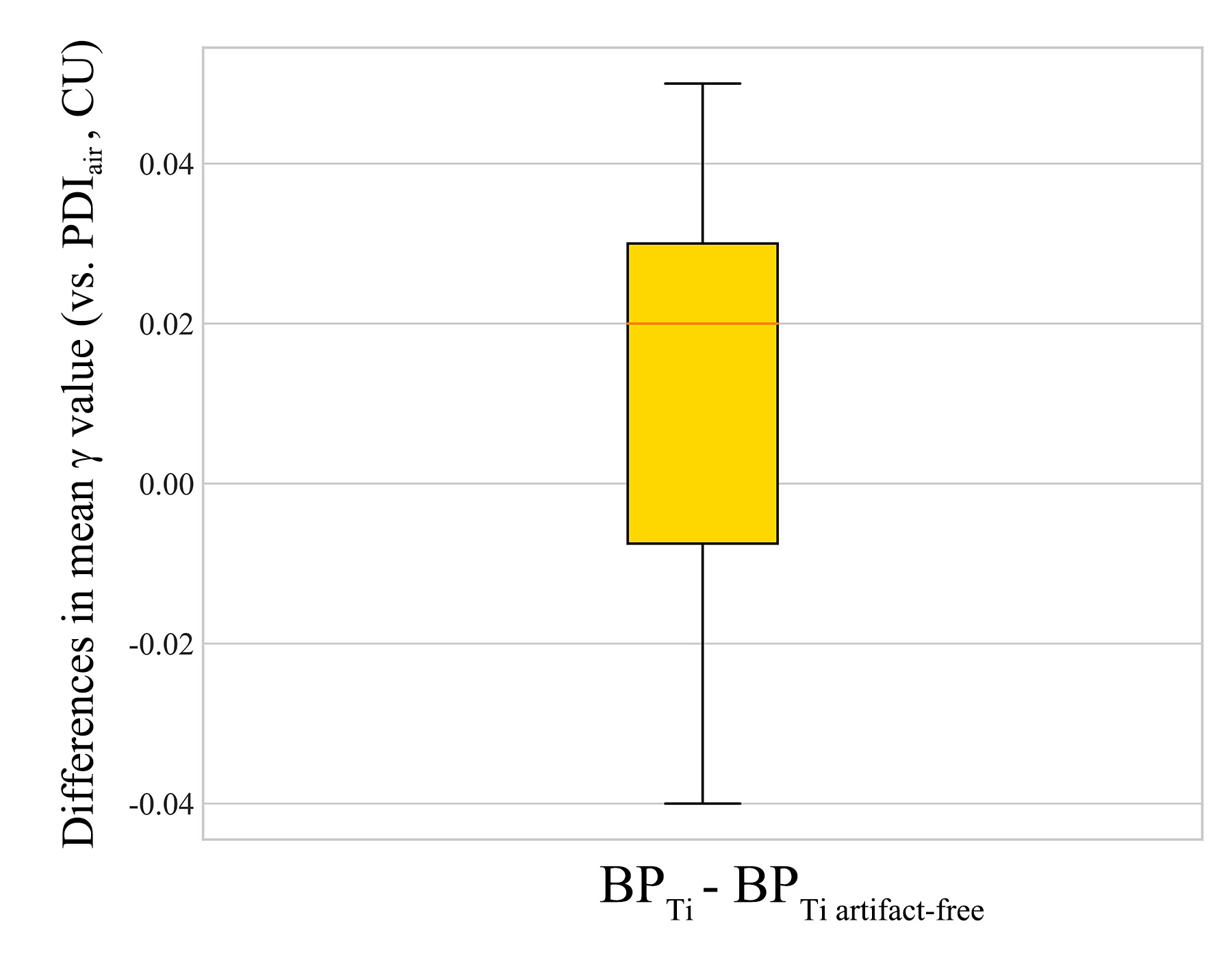

Supplement: Supplementary file 4 — Supporting Information [file ACM2-24-e14115-s002.jpg]

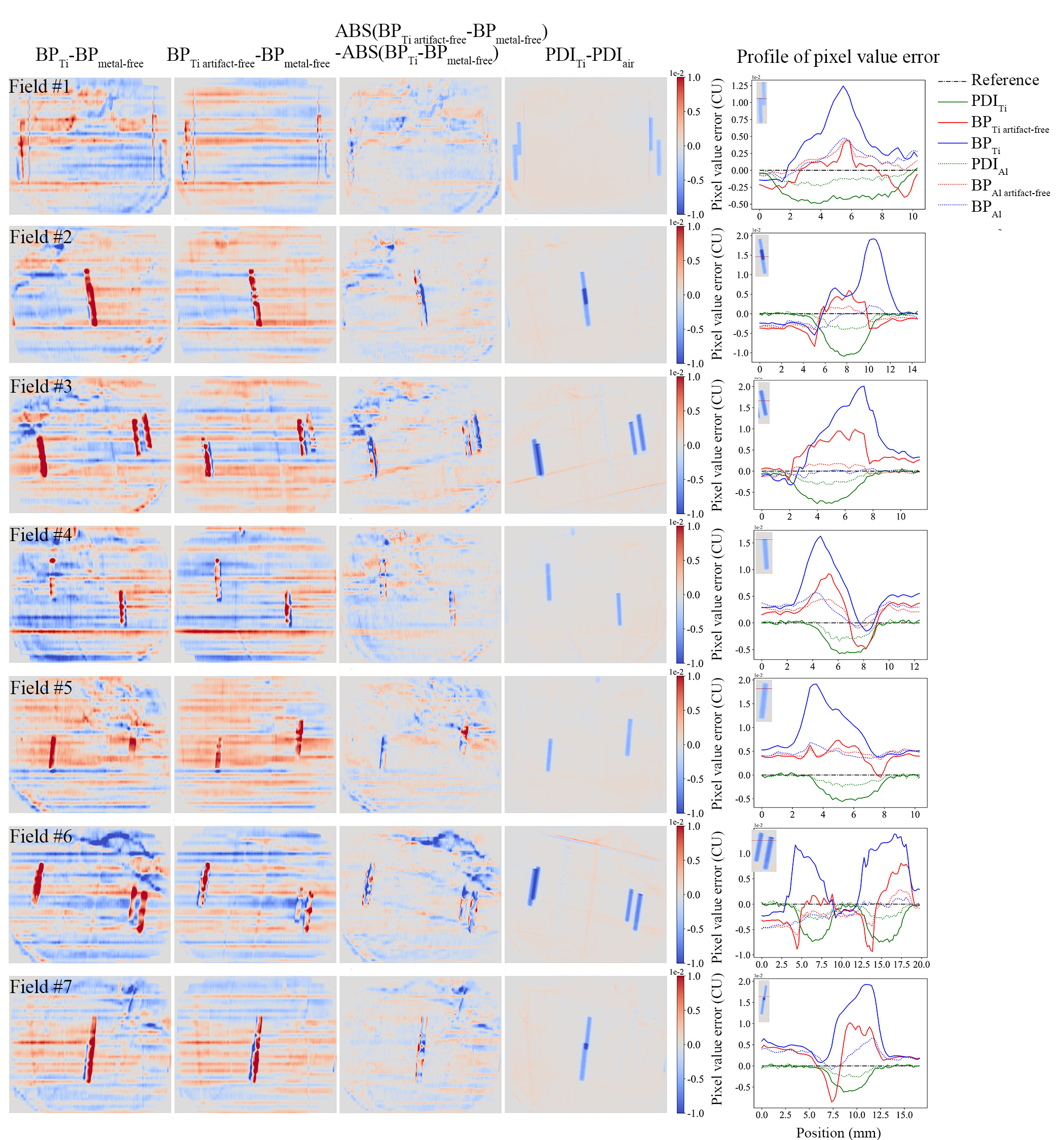

Supplement: Supplementary file 5 — Supporting Information [file ACM2-24-e14115-s003.jpg]

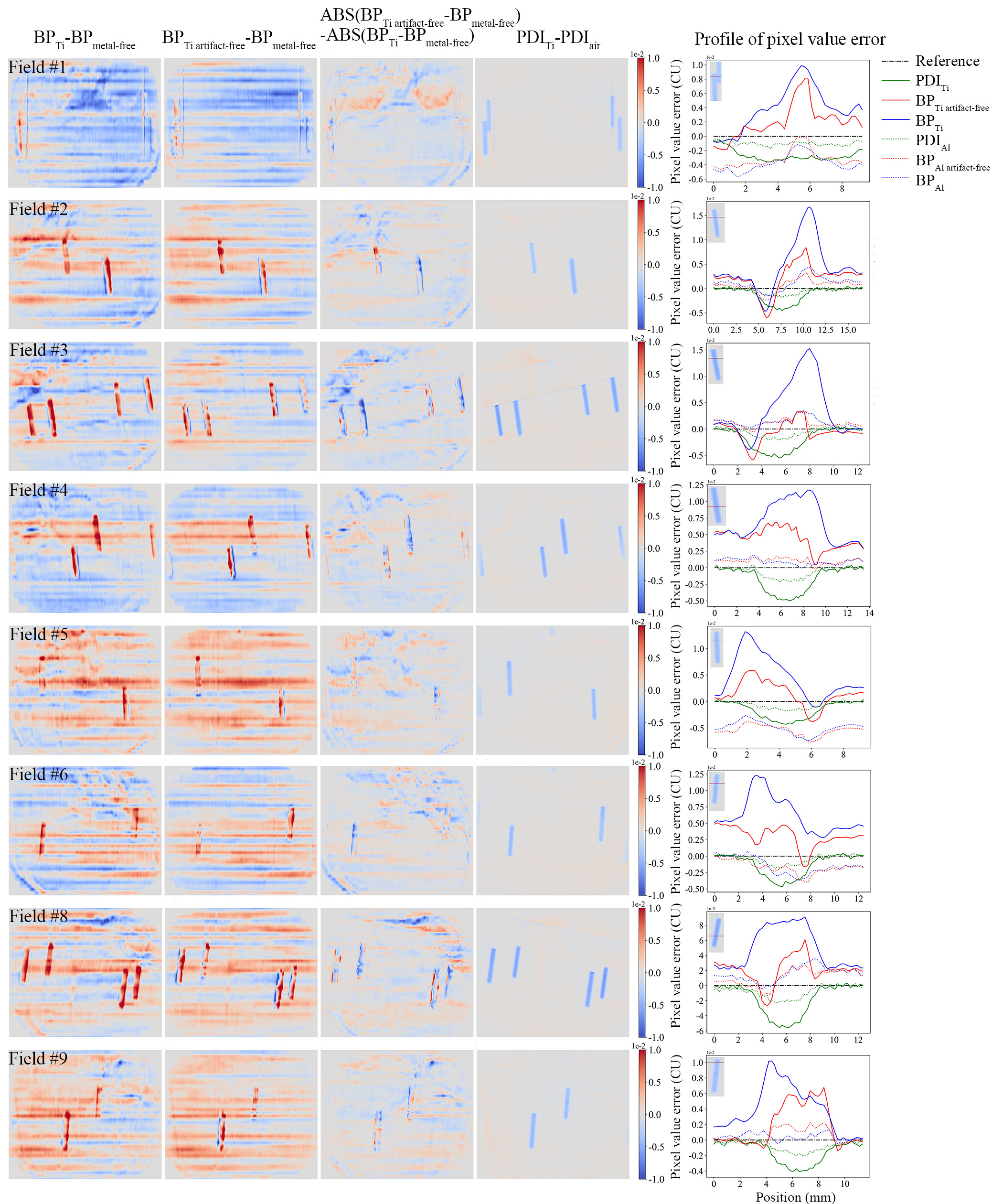

Supplement: Supplementary file 6 — Supporting Information [file ACM2-24-e14115-s008.jpg]

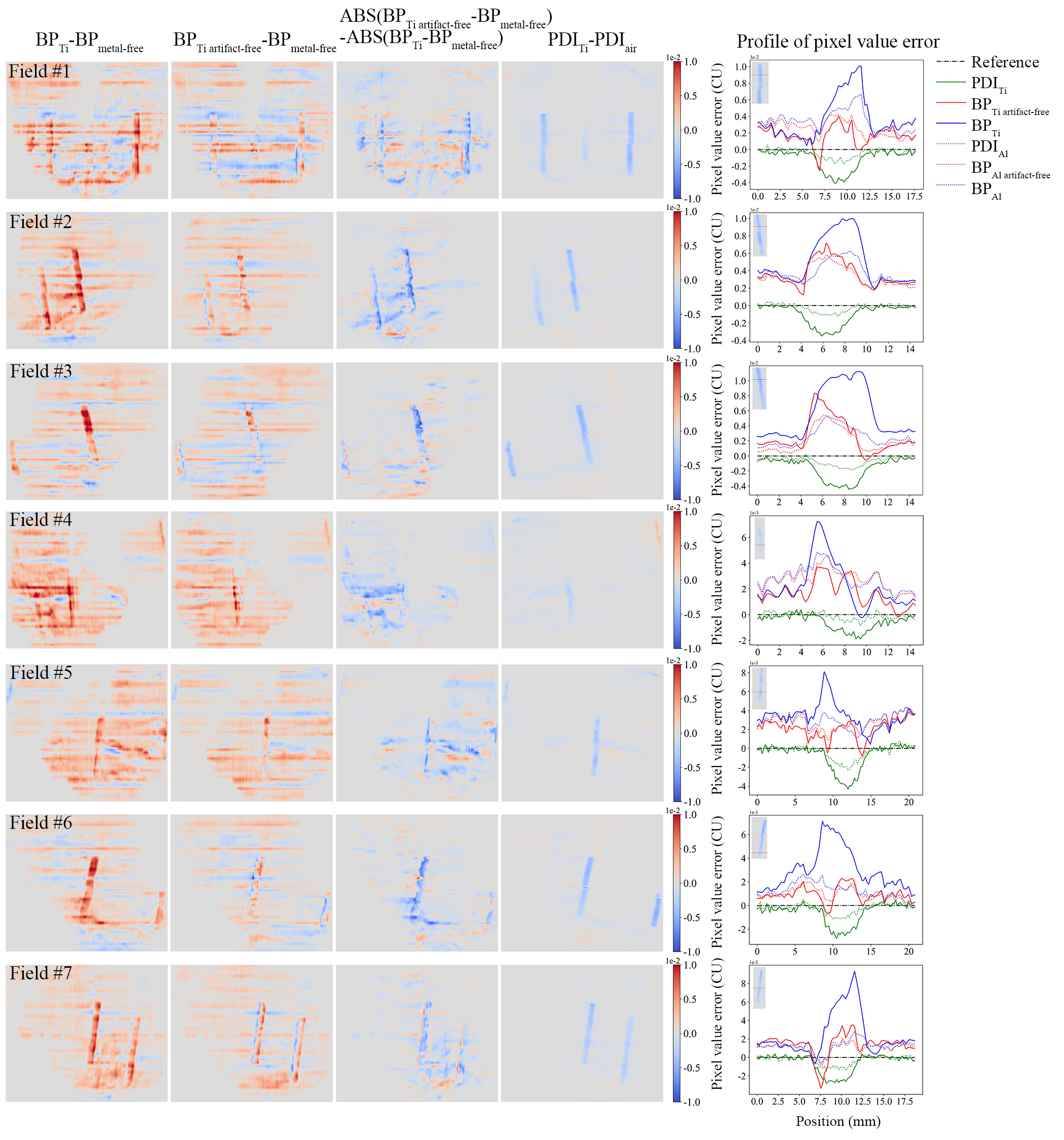

Supplement: Supplementary file 7 — Supporting Information [file ACM2-24-e14115-s006.jpg]

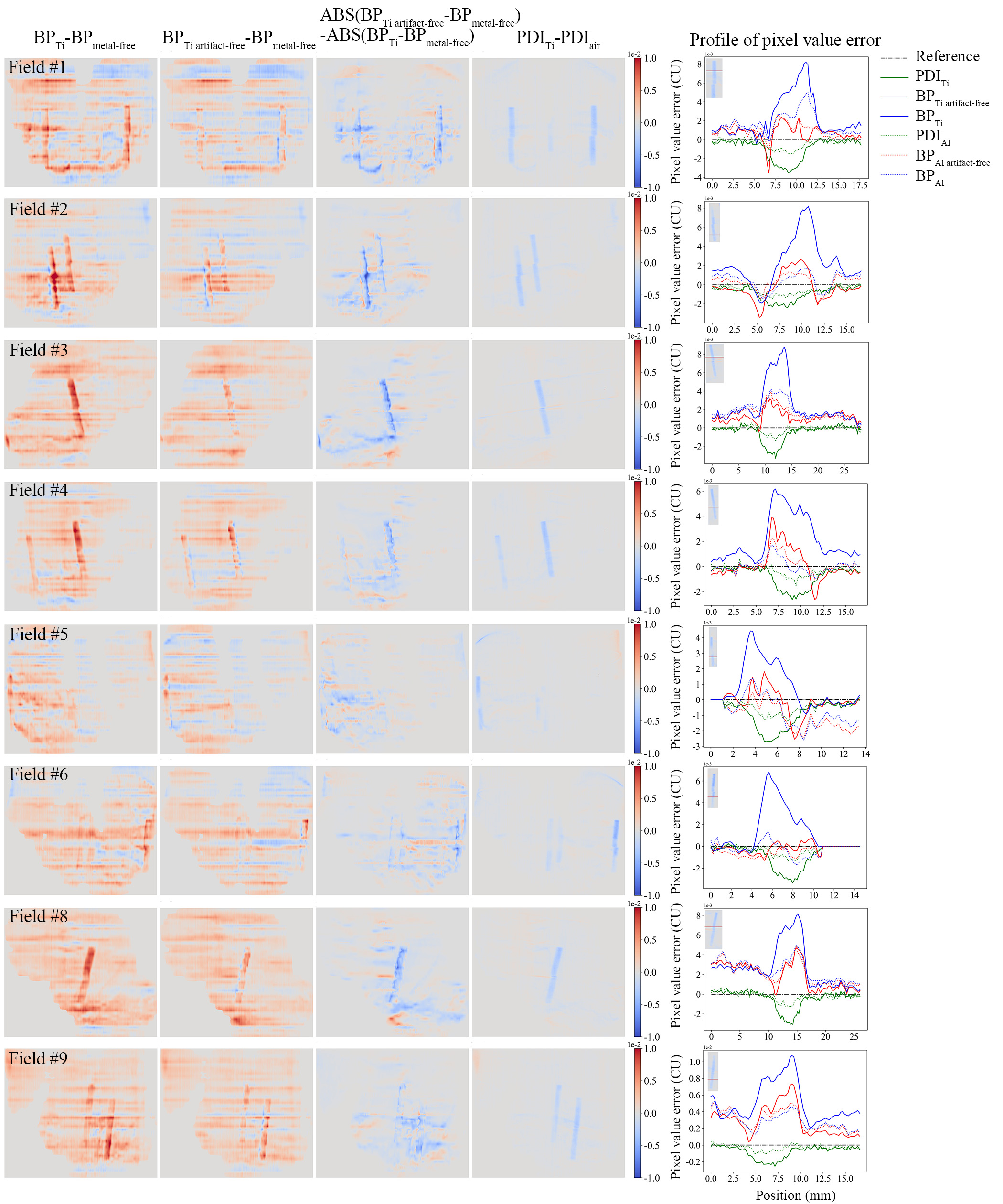

Supplement: Supplementary file 8 — Supporting Information [file ACM2-24-e14115-s001.jpg]
